# Optogenetic *in vivo *cell manipulation in KillerRed-expressing zebrafish transgenics

**DOI:** 10.1186/1471-213X-10-110

**Published:** 2010-11-02

**Authors:** Cathleen Teh, Dmitry M Chudakov, Kar-Lai Poon, Ilgar Z Mamedov, Jun-Yan Sek, Konstantin Shidlovsky, Sergey Lukyanov, Vladimir Korzh

**Affiliations:** 1 Cancer and Developmental Cell Biology Division, Institute of Molecular and Cell Biology, A-STAR, Singapore; 2 Shemiakin and Ovchinnikov Institute of Bioorganic Chemistry RAS, Moscow, Russia; 3 Department of Biological Sciences, National University of Singapore, Singapore

## Abstract

**Background:**

KillerRed (KR) is a novel photosensitizer that efficiently generates reactive oxygen species (ROS) in KR-expressing cells upon intense green or white light illumination *in vitro*, resulting in damage to their plasma membrane and cell death.

**Results:**

We report an *in vivo *modification of this technique using a fluorescent microscope and membrane-tagged KR (mem-KR)-expressing transgenic zebrafish. We generated several stable zebrafish *Tol2 *transposon-mediated enhancer-trap (ET) transgenic lines expressing mem-KR (SqKR series), and mapped the transposon insertion sites. As mem-KR accumulates on the cell membrane and/or Golgi, it highlights cell bodies and extensions, and reveals details of cellular morphology. The photodynamic property of KR made it possible to damage cells expressing this protein in a dose-dependent manner. As a proof-of-principle, two zebrafish transgenic lines were used to affect cell viability and function: SqKR2 expresses mem-KR in the hindbrain rhombomeres 3 and 5, and elsewhere; SqKR15 expresses mem-KR in the heart and elsewhere. Photobleaching of KR by intense light in the heart of SqKR15 embryos at lower levels caused a reduction in pumping efficiency of the heart and pericardial edema and at higher levels - in cell death in the hindbrain of SqKR2 and in the heart of SqKR15 embryos.

**Conclusions:**

An intense illumination of tissues expressing mem-KR affects cell viability and function in living zebrafish embryos. Hence, the zebrafish transgenics expressing mem-KR in a tissue-specific manner are useful tools for studying the biological effects of ROS.

## Background

The introduction of efficient transgenesis into the field of developmental biology opened the possibility to eradicate cells through the incorporation of tissue-specific and inducible toxic proteins [1-4], with cell death as an experimental endpoint. In addition, the ability to dose-dependently modulate the level of induced damage may be even more useful when investigating the long-term effects of experimental insult and/or recovery of affected cells. Fluorescent proteins not only faithfully report the presence of tagged proteins but, upon illumination, they also generate reactive oxygen species (ROS). The level of ROS generated can be modulated by a dose of illumination and evaluated by photobleaching of fluorescent proteins [5]. Different levels of ROS cause different effects: at low levels, ROS can promote cell division or differentiation; at intermediate levels - growth arrest; and at high levels - apoptosis. Hence, an intense illumination of fluorescent transgenic animals may, in principle, generate enough ROS to overcome the ability of cells to detoxify the reactive intermediates and thereby induce a state of oxidative stress. Overt production of ROS also damages the membrane and induces single strand breaks in the DNA. Probable biological outcomes, in increasing dose-dependent manner, are functional impairment, genetic instability resulting in somatic mutations or cell death [6-9].

Currently, reliable research tools to study the effects of ROS *in vivo*, that would enable both dose-dependent control and tissue specificity of ROS production, are not available. The green fluorescent protein (GFP) is mildly phototoxic under aerobic conditions, but since most vertebrates tolerate GFP phototoxicity, these toxic effects are low enough to be ignored [4,5]. In comparison, KillerRed (KR) is a much more potent photosensitizer, highly toxic, and efficiently produces ROS upon illumination [10,11]. Using purified KR and chemical probes to detect superoxide and singlet oxygen, it was shown that both types of ROS were produced upon green light irradiation of KR-expressing cells *in vitro *[12-15].

The semitransparent embryos of small teleosts, including zebrafish, are ideal for laser-mediated cell-ablation experiments [16]. We decided to explore the possibility of manipulating cells in dose-dependent manner in living, KR-expressing zebrafish embryos using widely available microscopes. Several stable transgenic lines, with tissue-specific expression of membrane-tethered KR (mem-KR), were made using the efficient *Tol2 *transposon-mediated enhancer trap transgenesis [17-19]. The KR-specific phototoxic effect in the CNS and heart of living vertebrates that we observed demonstrates for the first time the possibility to manipulate the viability and/or function of KR-expressing cells, and illustrates the utility of KR-expressing zebrafish transgenics as living tools to study the effects of ROS *in vivo*.

## Results

### The Tol2-KR screen

**Figure 1:**
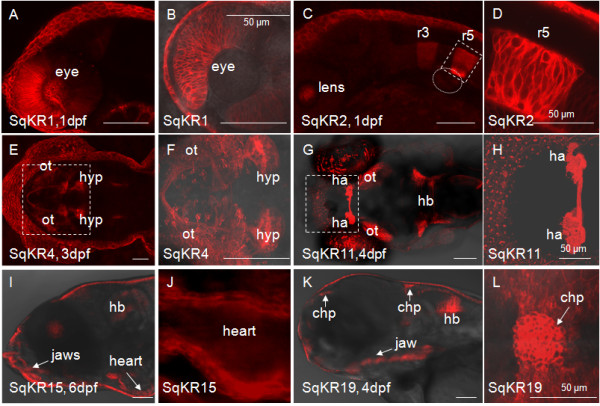
**Expression of the membrane-tethered KillerRed in some of enhancer trap transgenic lines**. (A-B) The head of SqKR1, lateral view. (B) The magnified view of the eye. (C-D) The head of SqKR2, lateral view. Mem-KR is expressed in rhombomeres 3 and 5 (r3 and r5). (D) A magnified view of the box in C. (E-F) The head of SqKR4, dorsal view. mem-KR is expressed in the optic tectum (ot) and hypothalamus (hyp). (F) A magnified view of the box in E. (G-H) The head of SqKR11, dorsal view. The habenula (ha), optic tectum (ot), hindbrain (hb) is highlighted in this projection. (H) A magnified view of the box in G enclosing the habenula. (I-J) The head of SqKR15, lateral view. (J) A magnified view of the heart, ventral view. (K-L) The head of SqKR19, lateral view. (L) Expression of mem-KR in the choroid plexus (chp) of SqKR19, dorsal view. All scale bars correspond to 100 μm, unless otherwise stated.

To test whether the KR would remain an efficient photosensitizer in developing zebrafish embryos, the cytosolic GFP reporter in the *Tol2 *transposon pBK-CMV enhancer trap (ET) vector [17], carrying a partial epithelial promoter of the *keratin4 *gene, was replaced by the membrane-tethered version of KR (mem-KR). A mix of the mem-KR (*Tol2-KR*) plasmid and transposase mRNA was injected into one-cell stage zebrafish embryos [17,18] to initiate random integration of *Tol2 *into the genome. Injected embryos were raised to sexual maturity when, as adults, they were outcrossed with wild type fish to identify transgenic progeny (F_1_) with tissue-specific expression of KR. These embryos were grown to maturity as founders of 20 families. Transposon integration site(s) were mapped using TAIL-PCR after crossing sexually mature transgenics with wild type fish. Expression patterns of KR in ET lines were documented by confocal microscopy (Figure 1). The integration sites and flanking genomic sequences of transgenics shown in Figure 1 are listed in Table 1 and 2, respectively. *Tol2*-mediated transposition into genomic DNA was defined by the presence of genomic sequences flanking *Tol2*. Not all KR-expressing transgenics resulted from the transposase-mediated transposition. For example, the insertion site in SqKR1 is flanked by *Tol2 *concatemer (Table 2) and represents a relatively rare event of random integration of the plasmid DNA into the zebrafish genome; i.e. the event that took place independent of Tol2 transposase activity [18].

**Table 1:** Transposon integration sites in transgenic lines depicted in Fig. 1.

Insertion name	Chromosome	Integration locus or nearest gene
KR1	ND	
KR2	22	22,076 bp downstream of *cirbp*
KR4	16	151354 bp of CT027703.23
KR11	6	13,480 bp upstream of stk35l^b^
KR15*	NA^a^,19^b^	NA^a ^48 bp upstream of exon1 of pard6gb^b^
KR19*	8^a^, NA^b^	32,151 bp downstream of ENSDARG00000078279^a ^NA^b^

^a ^and ^b ^represent two independent transposon insertion sites detected by TAIL-PCR.

**Table 2:** Flanking genomic sequences of Tol2 transposon insertions.

Insertion name	Flanking sequence reads from 5' or 3' transposon ends
KR1	Insertion of a concatemer of*Tol2*-containing plasmid

KR2*	5:**cct**ACAGCAGGGCCTTCACGTCACCTCTGATACCTCGCACGGTAGACCTCATTCCAGGAAAGACCAAGAGAGCGAACAGCTGCCCTCCTCCTTAAATCCCTAAGCCAAGTGCAGTCATACCTTCATATGCCCCCTTTGGACCAACACATTCAACCTTCCACCCACTCATGAAGGAATGGCGAAAGAGGGGTGAACGGGGATATCATGTAAAAATGCTGGAAGAGAATGATGTTATATCACGAAAATAAAAACGGCTGCAGAGAGGAAGGAAAAAGAAAAATTGGGGGGAAA
	3:**aggaaaag**AGCATAAACCTGCTTTAGACAGCTGTCGTTCGCCGGATCAGCTACTCAAGAGGCTGTCGTCTATTTGTTGGGCGCTAGATGTGAGCTGATCAATGGGAGACCCCTGCTGGTGTGTTTATGCAACTGCAGTGGCTTTGAAGAACTTAAGTGATCCAATGTACAATGTAGAGTGTGTTGTTGAATGGATGATATATATTTCAGTGCCTTTATTTATCAGGGGTCACCACAGTGGAATGAACCGCTAAATTATCCAGCATAGGTTTTTAC

KR4	5:**catggttt**TACACAGCTGATGGCCCTTCCAGCTGCAACCCAGTACGGGGGAAACACCTATACACTCATTCACACACACACACACACACACAC

KR11	3^a^:**attaattt**TTTCATTCATTTGGTTCAATTTGCCAAAATATTATTAAAATGTTACAAATTGCTTCCAAACACATCTACAACTAGCCTAAAAGGTTGATCACAGGACAAATAAGTCATAAATAGAACATAAGAAGGAGAAAATTATATTTCAATGGTTACCTGCTCTTTTGTCTATGAAGGTATTGTTGTCTCTTTGCTCAACTCACCTCTTTATGTCTTCAAATGACAGGGGTTCATTCACGGTACACTGACAGTCACATATAATCTTAACCAATGCACAGTCTAAGCTGATAGGAGCCATGATCGTTCGTTCATCACGTGACAGAATGACTTAAACCCAAGGACTCGAGAGATGAGTGGTTCAATTCTTTTTCCGGCTCGAAACGCGTATGATTGGCCCGTGATGAACGAGTGACTCAGACCCGATAGAGGACTCTAGAGGTGAGCGGATCAATTCTTTTTCCAGCTCTAAACGCATATGATTGGCTTCTGCCAATGCGATGAAGATTTGAAATTAACGATACTACCTGCACAAATGTGCGC
	3^b^:**gttttgca**GGCAACACTTGCTGAAAGCGGGGATCCGACAGAAGCGGAATTATGACTTGAAAACGAAAGGGCGACACTTCACAGCGGGAGAGAGGGTGTGGGTGTACAGCCCACAGAGGAAGAAGGGCAGGTGCCCTAAACTTGATGCACAGTGGGTGGGGCCTTGCATTGTACTGGAGAGAATTGGGGAAGTTGTTTACCGTGTGCAGATGCCACCAAGAGGCAGGAAAGTAGCGGTGCACAGGGATAGACTGGCTCCGTACAGAGGGTGTGCTTCAGCAGATACAGTGCTGGCCTCTCCTACAGTTATGCCTGTTCTGGAGAATGAGGCGGGACACGGGTTGGGTAGTTCTAATACTATAGATGTCCTCCTTGAAACTACACCAGTTCCTGAGCTACAAAGTTCACATTCTGGTGGCCCTGTTGCAGATAGGGGCTCACCTCGGTCTCAGAGAGTCAAGAGGCCACCAAGACGTCTCCAGGACTATGTTTGTTCCCTCGAGG

KR15	3^a^:**gttggtca**AATATTTGTTAGCTAGCCTATCATTAAAAAGCAAAGTTATTCTGCAATCAGGTTATGTGCTCCATCAGGCACAATCTTAATAGTATCTTGTCGTGTGCTATGCCTAAGTTTTCTTTGTCTTGTAGTGTGTGCACGTTTAAGGTTTGTGTGCACTGTAACCAGATTTCATTTTCGGTTGGGATTTTAAAACTCCTGCAGCAAGTTAGGAGAAAATGAAAGCACATTAAAATGAGGAAAAATCAAATGCTTATGACAATCTTTAACATGACAAATTAAAAATGTAGCAAACAAATGACGGGTTTGTGACCCAATTACAGTGGAGCATTAAAGAAACCCTTATTTCCCACAGAACAAAAAAAATCATCTATTCATGGGCAGGAACAAAAACATACACTTGTATCTTTCTTGTATCTTCATATTATGTGCAATATTTACATTTCTAGAGAAGCCTGTTTTAAACTGTTTTGATTATTACTGACATTTATCATTCATTGCGCTGGTCACTTTAAATGAGCCAGGGCAGGCTTTACAAGCTCTTTGCAGCACTGACCTTGTTCCCAGATCACTTCGGAAGAATGAACTTGGTTGGATGAATGA
	3^b^:**ctaatcag**TACCTCTCACTAGGGGGAGTGGGCTAAGTTTTCACATATGACATCATCGCTCTGTCTCCGACGGGTTGCTAATTCTACTACGGTGAGGGTTTGGCTTATGAATATTAAGTACGTAACGATGGTCTTCCTTAGAGGTAGCTGATTGGCTGCAAAGCGAACGTTAGTAAGCGGGATAAGGTGATGCGGTTGATTTCTTGATTAAAGGGACAGTCACATTTTTACATGCATAAACAAAGATGCATGGCTTCAATCCTAAATTTTCGACATTTTCAGATTGAAGTTGACGCTCAGCTCAAATCTTACAACAAAACAACAAAAATGTGTCCGAGTTTTGGTACTATATGAAGAACTGTCGCTGTTGATTCTGATTTGAGTAAGATAGCTTTTCTGTAATGCTCTTAATTTATTTATTTTCATACACTTTTGTTAAATTGTGATTTACTTACCCCCTTCTTTGTTTTGTTAGTCTTTCTCTCTCTG

KR19	5^a^:**gcacctca**CTGTACAGTAGGTGACATGACAATGTAAACAAAGAACTCCGAGATGAGCAGATAAACAAACCGTATCAGTCATTCAAACAAGCCTTATACTAAATGCTTAAATCGTGTTTGCTTTTTGAAAAGAGCCACTTACTTTTTGTCCAGGTAACTTAAGGTGGAAGATGTTACATTCCGTGGCACACAACTGTCCGGTACGAACAAAAACGGTCACCAAAGGCACATACACGCGCCAATGTCCATCTGTTCGTTCTTTCTCAGTTACCAAAGTTTCCAGTCGCCGCAGTTTATACAAAAGTACAGGCTAGCGTTCATAAGCCGGGTACTCTTTGAGAGAAAGAAGCTACGCAAAGCTCAAACAAACTTTCCCATTTCCAATCGCACTGTCATCCTTCACCATATCACGAGGGGAAAAATCCGTTAATATTCCAAAGTCAAACGCGCGCTCCCTCTCTGTACAAGTCCAGTCAAGTGTTTGCAGGTGTCTAGTGTGCTGTGTGCTGTACCTCGCCCTCTCTCCACTACCTCCCTATTAAAACCAGCCAGCCGTGTCAGTGTTAACCAGCTCCCTGCCAGCGCACATGCAACTGCG
	3^b^:**gctgtggc**GATCACAGATTAATAAAGGGACTAACCGAAAAGAAAATGAATGAATAACGCTGGATCTAACTTCGTTAACACACAAAAAACAGCTTGTTTTGATTATTGGTTTTGTTTTATTTTGCTTTTTGTCTGTTTTTTTTAGAATTTTATTTAAATTTAGGCAAAAATTTTCCCAGTGATGGGTTGCAGTTGGAAGGCCATCCGCTGCATAAAACATATGCTGGATAAGTTGGCAATTCATTCTGCTGTGGCGACCCCAGATTAATAAAGGGAATAAGCTGGAAAAAAACTAAAGAAAAATTGTTAGCTAAAATAGTCCACCCTGCCATCTAGGACTTTCTGTTTCTGTTTTTTCTTAAAGTTGTAGCATACCGCATGTAATCCATTATATTCAATGCTAATTTTTTTATGCTAAATGTAATTTTGAACAAAGCAATTGCAAACAAAAATGTATCACATTAATCCATGT

To obtain the genomic location of *Tol2 *insertions, the flanking sequence reads were Blast analyzed against the zebrafish genome sequence database (Zv8) http://www.ensembl.org. In some cases, the flanking sequence reads were Blast analyzed against the unfinished, high-throughput genomic sequence (htgs) database or trace archive at NCBI http://www.ncbi.nlm.nih.gov/projects/genome/seq/BlastGen/BlastGen.cgi?pid=9557. Sequences flanking Tol2 ends (not listed) are highlighted in bold.

### Photobleaching of mem-KR is associated with increased cytotoxicity

**Figure 2:**
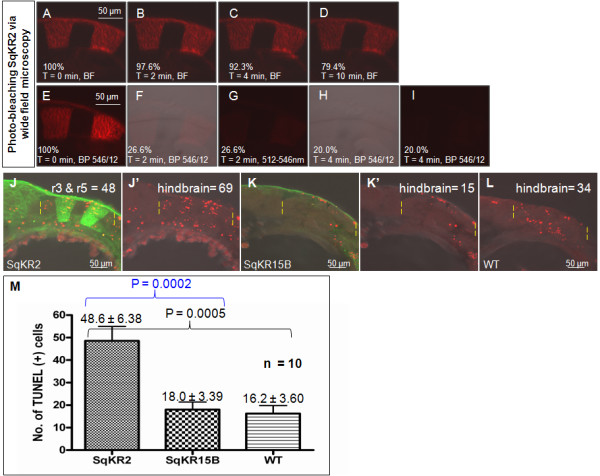
**Illumination of the hindbrain of SqKR2 caused bleaching of KR followed by increase in apoptosis**. (A-I) Efficient photobleaching of KR was achieved by intense green light using the UV lamp of the compound microscope in widefield mode. (A-C) Fluorescent merged images of the SqKR2 embryo at various time points, before and after illumination with white light. (E-I) Fluorescent merged images of the SqKR2 embryo at various time points, before and after illumination with green light. F and H are the bright field and fluorescent merged images of E and G, respectively. (J-L) Merged fluorescent/DIC images of KR-expressing cells (green) and TUNEL-positive cells (red) in SqKR2 (J), SqKR15B (K) and wild type zebrafish embryos (L). (J, L) Embryos were double stained for TUNEL (red) and (J, K) in addition by anti-KR antibody (green). (M) When compared to illuminated controls apoptosis increased more than two-fold in the hindbrain of illuminated SqKR2. A bar chart documenting the average number of TUNEL-positive cells per embryo in 10 embryos of three illuminated groups (SqKR2, SqKR15B and WT control). Values presented as mean ± SEM. Paired T test between the illuminated SqKR2 embryos and controls showed that the difference in the average number of TUNEL-positive cells is significant (P < 0.05). P values between groups are highlighted by the enclosing brackets.

We studied the extent of ROS-mediated photodamage associated with *in vivo *irradiation of 24 hpf SqKR2-transgenic embryos expressing mem-KR in rhombomeres (r) 3 and 5 (Figure 1C). Previous studies have shown that oxygen is required to elicit the photosensitizer properties of KR *in vitro*, and a surge in ROS production is associated with KR photobleaching [10-12]. Therefore, for illumination and imaging, embryos were mounted in uncapped, glass-bottom petri dishes (Mattek) in 1% low-melting point agarose, where diffusion provides sufficient oxygen to aerate the specimen. To compare the efficiency of different sources of light, KR-expressing embryos were exposed to maximum intensity of white (halogen 12 V/100 W lamp) or green light (UV filter set 15, BP 546/12 nm, the mercury arc lamp) from the Axiovert 200 M upright microscope. After 10 min exposure to white light, the SqKR2 embryo retained 79.4% of its fluorescence (Figure 2A-D). In contrast, after 4 min exposure to intense green light, the SqKR2 embryo retained only 20% of its fluorescence (Figure 2E-I). We next examined the effect of continuous exposure to the 1 mW HeNe543 laser at maximal intensity in confocal microscope image capture mode. Even after 80 min of such treatment, the SqKR2 embryo retained 51.1% of its fluorescence intensity (Additional file 1A-E). This demonstrated that KR photobleaching elicited by green light from the mercury lamp in the widefield mode is an efficient way of photobleaching KR in living zebrafish embryos.

We next compared the extent of KR photobleaching and the degree of damage to embryos by measuring the level of damage in DNA using the TUNEL assay on both illuminated control (wild type) and SqKR2 embryos. During this developmental period, some apoptosis is normally taking place resulting in some staining in controls. Since ROS are known to diffuse across membranes [20], their effect may spread outside of areas of KR expression. An exposure of KR-positive embryos to intense green light caused substantial increase in the number of cells detected by TUNEL assay: 4 min of exposure to green light resulted in a two-fold increase in the number of TUNEL-positive cells (Figure 2S) as compared to the control (Figure 2R). This demonstrated that *in vivo *photobleaching of KR causes cell death (Figure 2E-I; R-T). Since SqKR2 has both skin and rhombomere-specific expression, we next addressed the contribution of skin-specific mem-KR expression into cell death. Ten sets of 24 hpf embryos including SqKR2, SqKR15B (a line with basal skin expression obtained from SqKR15 outcross with wild type zebrafish) and wild type zebrafish embryos (used as negative control), were illuminated and fixed in 4% paraformaldehyde within three hours after illumination. Whole mount zebrafish TUNEL staining followed by immunohistochemistry to detect KR expression using anti-KillerRed antibody were carried out to address a question, whether there is an increase in cell death at the site of KR expression (Figure 2; Additional file 1F-K and Additional file 2). Only TUNEL-positive cells in the hindbrain were counted. An increase in cell death was detected at the site of KR expression in illuminated SqKR2 embryos (Figure 2J and Additional file 2). In fact 60% of apoptotic cells were found within KR-positive rhombomeres 3 and 5. The rest are either adjacent to these sites or represent background apoptosis. On average, the number of TUNEL-positive cells is approximately two-fold higher in SqKR2 (48.6 ± 6.38) when compared to both controls [SqKR15B (18.0 ± 3.39); WT (16.2 ± 3.60); Figure 2K-M]. Paired t-test comparing illuminated SqKR2 with the controls (SqKR15B or WT) further showed that obtained mean values are significantly different (P < 0.05; P = 0.0002 and P = 0.0005 respectively). In addition, no significant difference in the number of TUNEL- positive cells was detected between both controls (P = 0.6908). Thus cells expressing mem-KR are much more prone to illumination-induced DNA damage detected by TUNEL.

### KR-mediated heart damage

**Figure 3:**
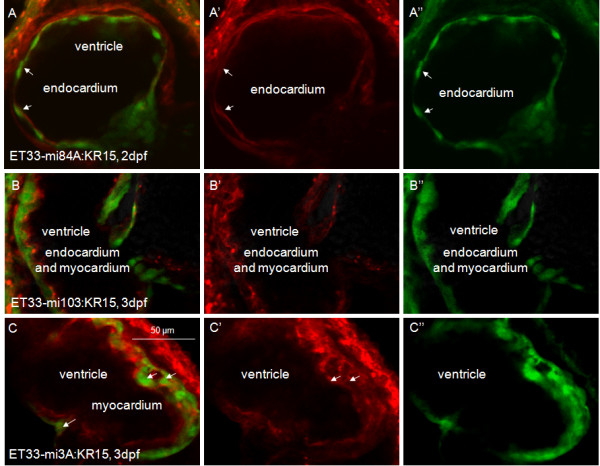
**KillerRed expression in SqKR15 is present in all layers of the heart**. (A) Co-localization of GFP and KR in the endocardium of the ET33-mi84A:SqKR15 double transgenic embryo (arrow - endocardium). (B) Co-localization of GFP and KR in the endocardium and myocardium of the ET33-mi103:SqKR15 double transgenic embryo. (C) Co-localization of GFP and KR in the myocardium of the ET33-mi3A:SqKR15 double transgenic embryo (arrow - myocardium). (A, B, C) merged images; (A', B', C') - KR expression; (A'', B'', C'') - GFP expression.

We next used SqKR15 embryos to assess effect of illumination on the heart (Figure 1I). The fate of KR-positive cells after illumination was followed in double transgenics of SqKR15 and cardiac enhancer trap (CET) transgenics expressing cytosolic GFP in the inner endocardium or outer myocardium [21,22]. Comparative morphological analysis of double transgenics of SqKR15 and SqET33-mi84A (endocardium), SqET33-mi103 (endocardium and myocardium) or SqET33-mi3A (myocardium) demonstrated that mem-KR enclosed cytosolic GFP-positive cells in both myocardium and endocardium layers (Figure 3), so the red and green signals are present in different subcellular domains.

**Figure 4:**
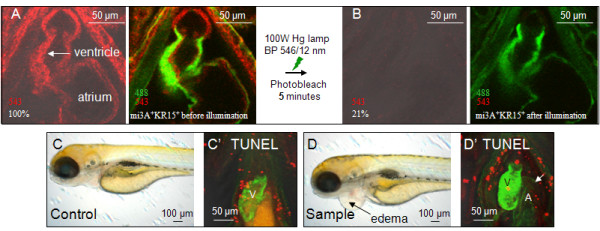
**Illumination of KR-expressing heart bleaches KR and causes cardiac edema**.(A-B) KR expression in the 3dpf beating heart of SqKR15 was reduced to 21% after 5 min of exposure to intense green light. (A) Expression of GFP and KR in the ET33-mi3A:SqKR15 double transgenic embryo prior to illumination and (B) after illumination. (C-D) Larvae at 4 dpf, one day after illumination: C - ET33-mi3A (control), D - ET33-mi3A:SqKR15 (experimental sample). Cardiac edema developed in ET33-mi3A:SqKR15 larvae one day after illumination (D). Apoptosis in the heart of SqKR15 is similar to that in control.

Previous studies have shown that oxidative stress is one of the factors linked to cardiovascular disease and heart failure [23,24]. Given that photobleaching of KR efficiently produces ROS [10-12] availability of transgenic zebrafish expressing KR in the heart created a possibility to study the effect of ROS on the heart of vertebrate larvae *in vivo*. Confocal imaging of SqKR15/SqET33-mi3A 3dpf larvae before and after 5 min exposure of KR-expressing heart (atrium and ventricle) to intense green light showed that bleaching of mem-KR in the heart (21%) does not affect cardiomyocyte-specific GFP expression (Figure 4A-B). One day later all illuminated SqKR15/SqET33-mi3A larvae developed pericardial edema (Figure 4D, n = 3; Additional file 3A-C); this represents a special case of fluid overload. In humans, this condition, which is common when cardiac output and circulation are insufficient, manifests as a form of pulmonary congestion [25]. Since the larvae do not have lungs and the gills are not developed as yet, an accumulation of fluid occurs in the pericardium. Despite the presence of edema in the pericardium, no obvious increase in TUNEL-positive cells (Figure 4C'-D') was observed a day after illumination in the heart of illuminated SqKR15/SqET33-mi3A larvae. The only defect is the visibly distended GFP (+) atrial myocardium (Figure 4D'; white arrow).

**Figure 5:**
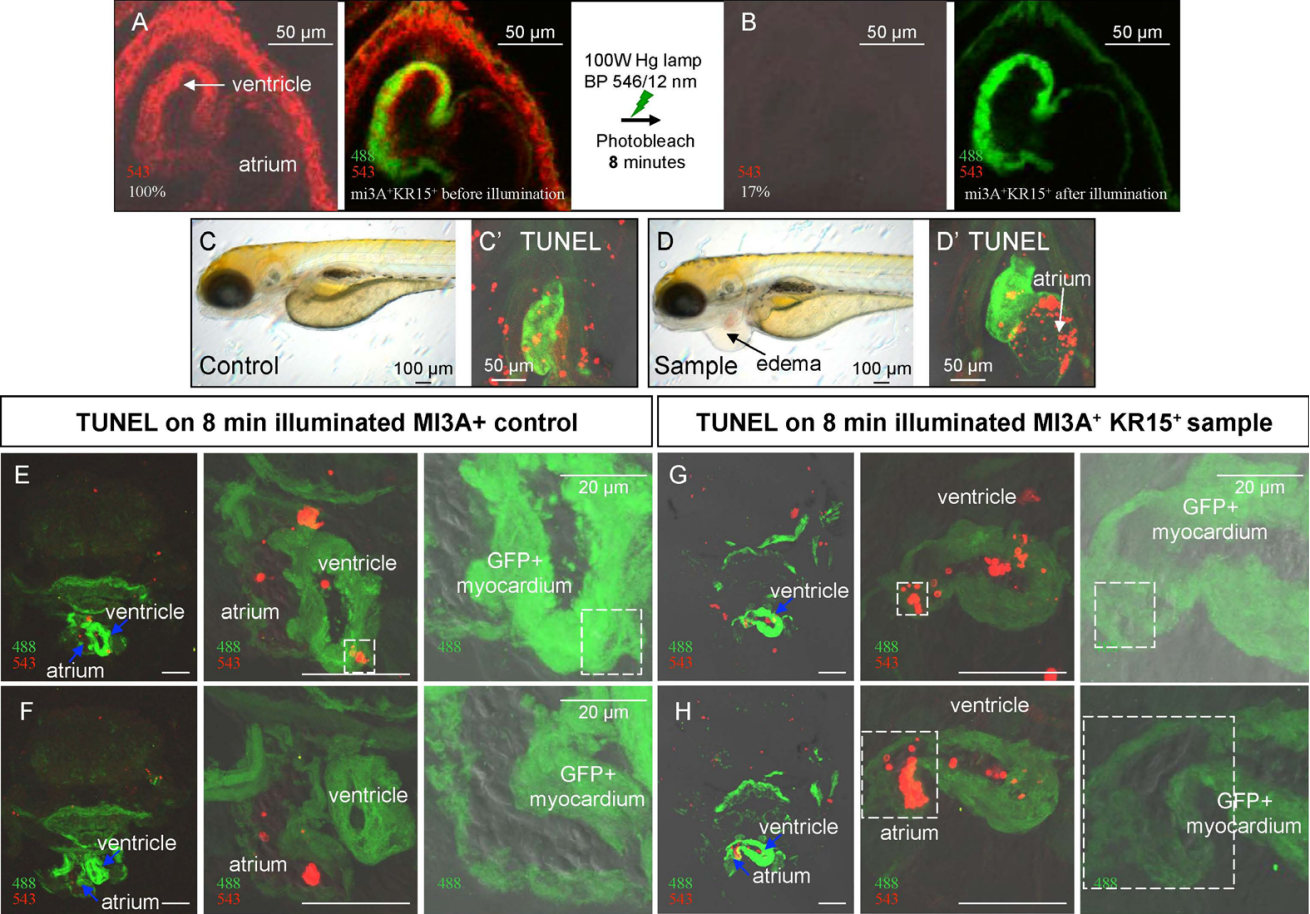
**Increased illumination increased apoptosis in the KR-expressing heart**. (A-B) 8 min illumination of the 3dpf heart of SqKR15 embryo with intense green light reduced fluorescence intensity to 17%. (A) GFP and SqKR15 fluorescence in the ET33-mi3A:SqKR15 double transgenic embryo before and (B) after illumination. (C-D) same larvae one day after illumination: C - ET33-mi3A (control), D - SqKR15/ET33-mi3A (sample). TUNEL (+) cells in the heart of illuminated sample (D') and control (C'), a day after illumination. (E-F) TUNEL staining of transverse sections of SqET33-mi3A larva (control) and (G-H) SqKR15/ET33-mi3A larva (sample), at different magnification one day after illumination. Examples of TUNEL (+) cells in the myocardial layer are boxed in white. All scale bars are 50 μm in length unless otherwise stated.

In order to show that effect of illumination was dose-dependent, larvae were exposed to 8 min illumination by intense green light resulting in a further reduction of mem-KR fluorescence (17%) in the heart. Again, pericardial edema developed a day after illumination SqKR15/SqET33-mi3A larvae (Figure 5A-D; n = 6; Additional file 3D-I). In addition to a visibly distended GFP (+) atrial myocardium, enhanced apoptosis was also observed in this layer (Figure 5G-H; boxed in white). Hence prolonged illumination caused irreversible pathological damage. This was never observed after illumination of wild type control (Figure 5C, E-F).

**Figure 6:**
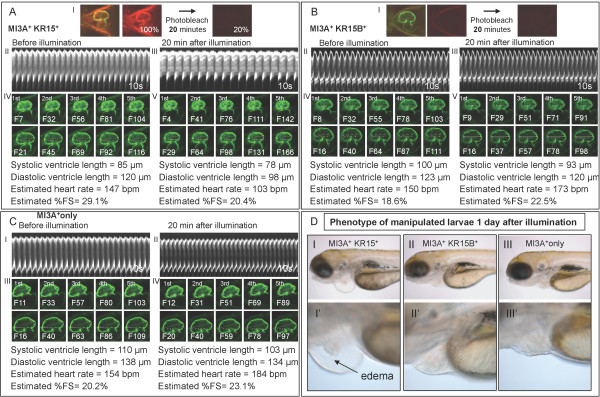
**A reduction in cardiac output was observed in KR-expressing larvae immediately after illumination**. (A-C) Heartbeat and contractility in SqKR15/ET33-mi3A (A), SqKR15B/ET33-mi3A (B) and KR-negative Sq ET33-mi3A (C) 3dpf larvae before illumination and 20 min after illumination. Panel (I) in (A-B) shows confocal images of corresponding double transgenic larvae, taken at the same gain setting, before and after illumination. M-modes depicting a heartbeat for 10 sec before and after illumination are in panels A-B, II-III and C, I-II. Images of five consecutive ventricular systole and diastole are shown in A-B, IV-V and C, III-IV. Cardiac output was specifically reduced after illumination of SqKR15/ET33-mi3A larva as the reduction in heartbeat is accompanied by a decrease in contractility indicated by a decrease in value of %FS. (D) Larvae at 4dpf, one day after illumination: (I) - SqKR15/ET33-mi3A (sample), (II) - SqKR15B/ET33-mi3A with skin mem-KR expression as a positive control and (III) - SqET33-mi3A as a negative control. Only SqKR15/ET33-mi3 developed prominent cardiac edema next day after illumination [D (I-I')].

To document changes in heart contractility, we recorded heartbeat using LSM 5 *LIVE *scanning microscope with continuous image acquisition at 60 confocal images per second for 30 seconds. M-mode depicting vertical movement of the heart tube edges (y axis) over time (x-axis) was generated [26] immediately before and after illumination. The effect of illumination on pumping efficiency of the heart was compared across each group (Figure 6A-C; Additional files 4, 5, 6 and 7). In total 5 embryos in each group were analyzed.

**Figure 7:**
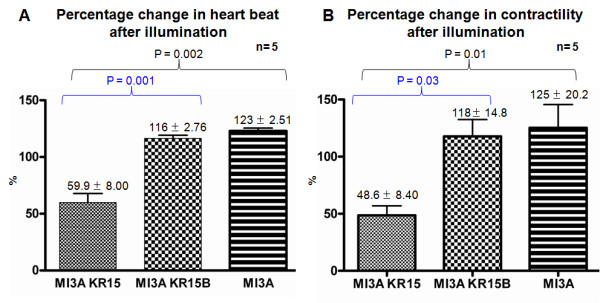
**Cardiac output is consistently reduced in all illuminated SqKR15 larvae**. (A-B) A bar chart comparing the percentage change in heartbeat (A) and contractility (B) after illumination of SqKR15/ET33-mi3A (sample) and controls (SqKR15B/ET33-mi3A; SqET33-mi3A) across three illuminated groups of five embryos each. Values presented are mean % change in heartbeat ± SEM (A) or mean % change in contractility ± SEM (B), where 100% indicates no change in heart beat or contractility after illumination. Paired T test between illuminated SqKR15 larvae and controls showed that the difference in value is significant (P < 0.05). P values between groups are highlighted by the enclosing brackets.

In all illuminated embryos a heartbeat and contractility were measured before and after illumination. On average, a 20% increase in heartbeat and heart contractility was observed in all illuminated controls (SqKR15B^+ ^MI3A^+^; MI3A^+ ^only; n = 5) immediately after illumination (Figure 6B, C; Figure 7A-B). We attributed this to a light-induced stress response to illumination. No adverse morphological changes developed in these controls a day after illumination (Figure 6DII-III; Additional files 4, 5, 6 and 7). In contrast, illumination of SqKR15/SqET33-mi3A larvae caused a reverse effect - decrease in the rate of heartbeat and heart contractility (with the latter being defined by the %FS value, Figure 6A). A day after illumination pericardial edema developed in all illuminated SqKR15/SqET33-mi3A larvae [Figure 6D (I-I'); Additional files 4, 5, 6 and 7]. On average a 40% decrease in heartbeat and a 50% reduction in contractility were observed immediately after illuminating SqKR15/SqET33-mi3A larvae (Figure 7A-B). Paired t test further demonstrated that the percentage decrease in heartbeat and contractility is significantly different from that in controls (P < 0.05; Figure 7) in absence of significant difference between the controls. The decrease in cardiac function observed immediately after illumination of SqKR15/SqET33-mi3A larvae suggested an immediate effect of KR-induced ROS production on heartbeat and contractility. Since the positive control with basal KR expression in the skin (SqKR15B) did not develop edema, the decrease in cardiac output is attributed to KR expression in the heart.

## Discussion

Optogenetic cell ablation is a promising approach for photodynamic therapy [14,27]. Since the mem-KR is less efficient in eliciting cell death than its histone-tethered version [9], it may be more applicable for experiments aiming to affect cell physiology through the negative influence of KR-induced ROS production, for example on the heart rhythm and contractility. In addition, mem-KR could be a useful tool to study the effect of ROS at sub-lethal levels linked to most forms of heart disease, including ischemia and sudden heart failure [23,24].

Here, the embryos of ET mem-KR transgenics were used for optogenetic manipulation of cell viability and function *in vivo *via dose-dependent, ROS-induced photodamage through the use of a commonly available mercury lamp rather than more specialized equipment, such as a laser, etc. We noted that mem-KR is more efficient as photosensitizer after illumination with the mercury lamp comparing to that of the laser of confocal microscope. This is attributed to the overall low dose of laser illumination and the fact that the 543 nm HeNe laser line used here is not optimal for KR excitation as the excitation maxima of this photosensitizer is at 585 nm. Thus, the confocal microscopy with the 543 nm HeNe laser line could be used to document mem-KR expression and cellular morphology before and after a surge of ROS production that can be conveniently induced by the mercury lamp attached to the same microscope. In a parallel study we have found that some specialized populations of KR-expressing cells are rather sensitive to illumination (Go et al., unpublished). Thus one needs to study the dose-dependent effects of laser/mercury lamp illumination in respect of a cell type under the study as these may vary.

The scale of photodamage associated with KR-activated ROS depends on various experimental conditions, such as cell type, illumination, oxygenation, and the availability of antioxidants [14]. The extent of KR bleaching after illumination could be a good indicator of KR-induced cytotoxicity. In our hands, 80% reduction of the mean fluorescence intensity of mem-KR, as quantified using Image J, consistently caused cytotoxicity (Figure 2, 5). Other factors affecting the degree of photodamage include tissue transparency, which decreases as development progresses, and the intensity of KR expression; the latter parameter depends on many factors, such as a distance of the insertion site from the enhancer [18], as well as the efficiency of the basic promoter used. The use of *krt4 *basic promoter often results in transgenics with relatively bright expression of fluorescent markers [17,18,21], which is probably due to the compatibility between this promoter with various enhancers and the high efficiency of their interactions [28]. The use of *krt4 *basic promoter-based enhancer trap system thus enhanced the chance of intense transgene expression, which could be important for applications based on relatively weak photosensitizers, such as mem-KR. Finally, the dose of illumination must be optimized for each transgenic line/tissue to elicit the KR-mediated photodamage at the desired level.

## Conclusions

In summary, the KR-expressing transgenic lines represent useful tools to study the effects of ROS-mediated injury in different living cell lineages, in a dose-dependent manner. Notably, the decreased cardiac output and subsequent pericardial edema that was induced by KR-mediated ROS production in the heart generated a phenotype that closely mimics the pathological condition associated with heart failure in humans. There is accumulating evidence to support a role for ROS in the development and progression of heart failure. Hence, KR transgenics may find their application in re-constructing the multistage processes caused by oxidative stress-induced damage in development and disease.

## Methods

### Fish maintenance

#### Zebrafish care and maintenance

Wild type (AB), cardiac enhancer trap lines [21] and ET (krt4-mem-KR) zebrafish lines were maintained in the IMCB zebrafish facility according to the IACUC rules (the Biopolis IACUC application #050096) and established protocols [29]. All experiments involving zebrafish embryos/larvae were carried out in accordance to IACUC rules. Embryos were staged as described [30] in hours post fertilization (hpf). Embryos older than 30 hpf were first treated with 1-phenyl-2-thiourea at 18 hpf to prevent formation of melanin.

### Molecular cloning and the generation of ET(krt4-mem-KR) lines

The KillerRed reporter-based Tol2 transposon pBK-CMV enhancer trap vector is a modification of the original GFP reporter-based system [17]. The GFP reporter flanked by BamH1 at the 5' end and Not1 at its 3' end was subsequently replaced by the KR reporter flanked by the same restriction enzymes. To make the mem-KR the membrane localization signal (MLS) of neuromodulin was linked to the N-terminus of KR. The MLS (N-terminal 20 amino acid residues of Gap43/neuromodulin) contains a signal for posttranslational palmitoylation of cysteines 3 and 4 that targets KR to cellular membranes [12,31]. Putative founders of the KR-expressing ET lines were generated as stated [17] by co-injection of transposase mRNA and the KR reporter-based Tol2 transposon pBK-CMV enhancer trap (ET) construct into 1-4 cell stage zebrafish embryos. The microinjected embryos were grown to maturity when each putative founder was out-crossed with wild type zebrafish and KR-expressing embryos were raised to adulthood resulting in F_1 _of the KR ET line. Three KR-expressing ET lines, SqKR2, SqKR15 and SqKR15B are emphasized in this article. SqKR15B, a line with basal KR expression in the skin segregated after outcross of SqKR15.

### Optical setup and embryo staining

For illumination and imaging of KR-positive transgenic zebrafish embryos, we employed an upright (Zeiss Axiovert200M) laser scanning microscope (LSM) Meta 510 (Carl Zeiss) equipped with a x40 numerical aperture (NA) 0.75 W Achroplan long working distance dipping objective, 100 W mercury lamp and two laser lines (30 mW Argon and 1 mW HeNe). Depending on tissue type and the age of the embryo, the x40 objective and continuous exposure to white (halogen 12 V/ 100 W lamp) or green light (4 - 10 min) from the 100 W mercury lamp and filter set 15 (BP 546/12 nm) were employed at maximal light intensity and objective aperture. Heartbeat recordings were acquired on an inverted LSM 5 *LIVE *laser scanning microscope, using the EC Plan-Neofluar 20×/0.5 Ph2 M27 objective at 28°C. Images were acquired at 60 frame/sec (512 by 512 pixels). Green light illumination was performed on the same microscope by 20 min exposure to light from the 100 W mercury lamp using filter set 15 (BP 546/12 nm), with the EC Plan-Neofluar 40×/0.75 M27 objective followed by the use of 20× objective to record 30 seconds of heartbeat after illumination.

The anti-KR antibody (Evrogen, Russia; Cat. No. AB961-AB962) and TUNEL kit TMR Red (Roche, USA; Cat. No. 12156792910) were used for two-color staining for KR expression and apoptotic cells, correspondingly.

### Data analysis

To record changes in fluorescence intensity the same gain settings were used for all images. Mean fluorescence intensity, before and after photobleaching in each frame was then compared and measured using the ImageJ freeware. The decrease in fluorescence intensity after photobleaching is presented as a percentage of the original mean fluorescence intensity. TUNEL-positive cells in SqKR2 data set were quantified using the count tool in Adobe Photoshop CS4. Images of the beating heart acquired using LSM 5 *LIVE *laser scanning microscope were opened in ImageJ, converted to AVI format and then processed to M-mode [26]. Measurements of heart contractility involved assessing systolic and diastolic ventricle lengths, using the ruler tool in LSM image browser (Carl Zeiss, Germany), before and after illumination. The percent of fractional shortening (%FS) is derived using the formula:

$$\%\text{FS}=\frac{\text{Diastolic diameter}-\text{Systolic diameter}}{\text{Diastolic diameter}}\times 100$$

Each heartbeat is defined as the time taken to complete one cycle of maximal dilation and contraction. The heart rate in beats per minute (bpm) from each data set was estimated by noting the number of frames required to complete four heartbeats. There are 3600 frames captured per minute. The heart rate (heartbeat per minute; bpm) is then calculated by the following formula:-

$$\begin{array}{c} \text{Heartbeat/min}=\frac{\text{Number of frames per minute}}{\text{Number of frames per heartbeat}}\\ =\frac{\text{(}60\text{ frames per second)}\times 60\text{ second}}{\left(\frac{{\text{Frame Number}}_{5\text{th systole}}-{\text{Frame Number}}_{1\text{st systole}}+1}{4}\right)} \end{array}$$

Column statistics and paired t tests were conducted using GraphPad Prism software.

## Authors' contributions

CT, KS, SL, VK developed the concept for this study. DMC and IZM generated the KR-*Tol2 *construct. CT generated the mem-KR ET transgenics and analyzed them with JYS and VK, performed and analyzed KR effect on zebrafish and with KLP analyzed expression of fluorescent proteins and effect of illumination of single and double heart transgenics. CT and VK wrote the manuscript. All authors read and approved the final manuscript.

## Supplementary Material

Additional file 1 **Illumination by the confocal microscope laser is inefficient in causing apoptosis**. (A-E) Changes in fluorescence intensity of SqKR2 embryo during 80 minutes of continuous confocal imaging. (F-K) Illumination by green light of mercury lamp in the widefield mode increases apoptosis in the hindbrain of the SqKR2 embryo (G, J). Relatively few apoptotic cells in the SqKR2 embryo were detected following continuous confocal imaging (H, K). The otic vesicle is defined by yellow broken line (Figure 2F-H). Click here for file

Additional file 2 **Compilation of TUNEL staining data used to generate the bar chart for apoptosis in the hindbrain**. (1-9A) Merged fluorescent/DIC images of KR expressing cells (green) and TUNEL-positive cells (red) in SqKR2 (A), Sq15B (1-9B) and wild type zebrafish embryos (1-9C). TUNEL-positive cells in each data set were quantified using the count tool in Adobe Photoshop CS4. Click here for file

Additional file 3 **Compilation of images of all illuminated SqKR15 larvae with pericardial edema a day after illumination**. Click here for file

Additional file 4 **First group of images used to generate the bar chart of percent change in heartbeat and contractility after illumination**. Heart beat and contractility in SqKR15/ET33-mi3A (sample), SqKR15B/ET33-mi3A (skin control) and KR-negative Sq ET33-mi3A (negative control) 3dpf larvae before and 20 min after illumination. Click here for file

Additional file 5 **Second group of images used to generate the bar chart of percent change in heartbeat and contractility after illumination**. Heart beat and contractility in SqKR15/ET33-mi3A (sample), SqKR15B/ET33-mi3A (skin control) and KR-negative Sq ET33-mi3A (negative control) 3dpf larvae before and 20 min after illumination. Click here for file

Additional file 6 **Third group of images used to generate the bar chart of percent change in heartbeat and contractility after illumination**. Heart beat and contractility in SqKR15/ET33-mi3A (sample), SqKR15B/ET33-mi3A (skin control) and KR-negative Sq ET33-mi3A (negative control) 3dpf larvae before and 20 min after illumination. Click here for file

Additional file 7 **Fourth group of images used to generate the bar chart of percent change in heartbeat and contractility after illumination**. Heart beat and contractility in SqKR15/ET33-mi3A (sample), SqKR15B/ET33-mi3A (skin control) and KR-negative Sq ET33-mi3A (negative control) 3dpf larvae before and 20 min after illumination. Click here for file
