# Supplementary figures and images for: Optogenetic in vivo cell manipulation in KillerRed-expressing zebrafish transgenics
Source: BMC Dev Biol. 2010 Nov 2;10:110. doi: 10.1186/1471-213X-10-110 (PMC2989954; doi:10.1186/1471-213X-10-110)

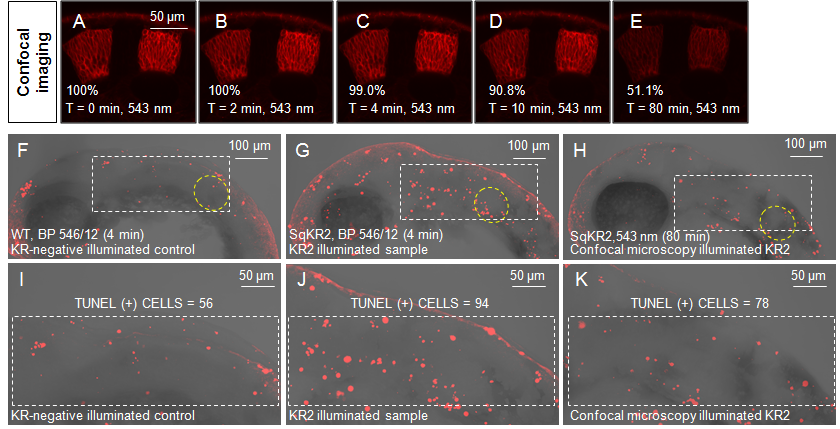

Supplement: Additional file 1 — Illumination by the confocal microscope laser is inefficient in causing apoptosis. (A-E) Changes in fluorescence intensity of SqKR2 embryo during 80 minutes of continuous confocal imaging. (F-K) Illumination by green light of mercury lamp in the widefield mode increases apoptosis in the hindbrain of the SqKR2 embryo (G, J). Relatively few apoptotic cells in the SqKR2 embryo were detected following continuous confocal imaging (H, K). The otic vesicle is defined by yellow broken line (Figure 2F-H). [file 1471-213X-10-110-S1.TIFF]

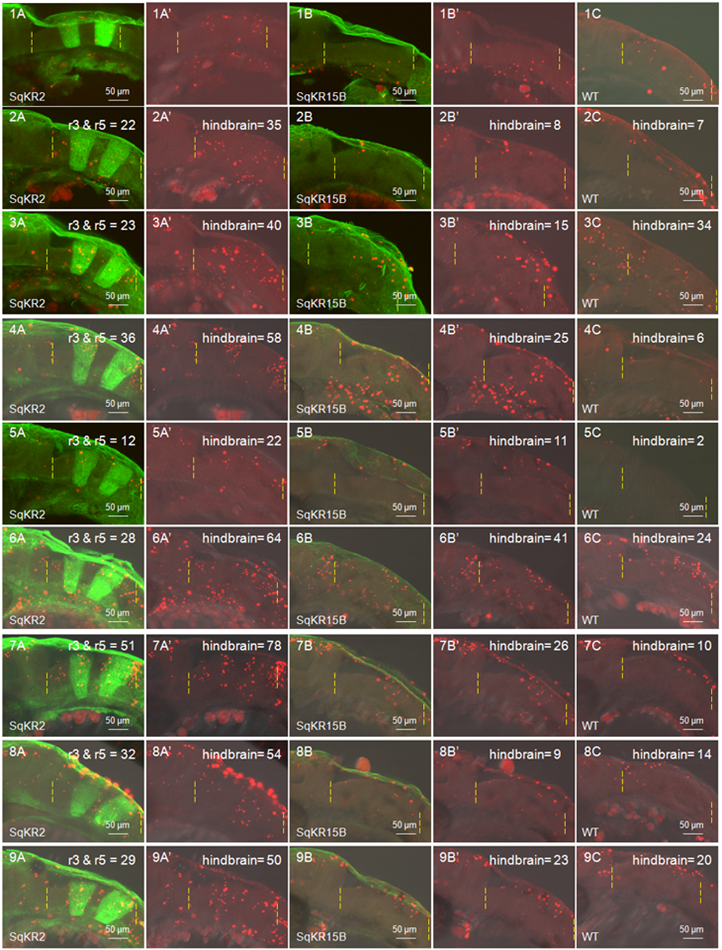

Supplement: Additional file 2 — Compilation of TUNEL staining data used to generate the bar chart for apoptosis in the hindbrain. (1-9A) Merged fluorescent/DIC images of KR expressing cells (green) and TUNEL-positive cells (red) in SqKR2 (A), Sq15B (1-9B) and wild type zebrafish embryos (1-9C). TUNEL-positive cells in each data set were quantified using the count tool in Adobe Photoshop CS4. [file 1471-213X-10-110-S2.TIFF]

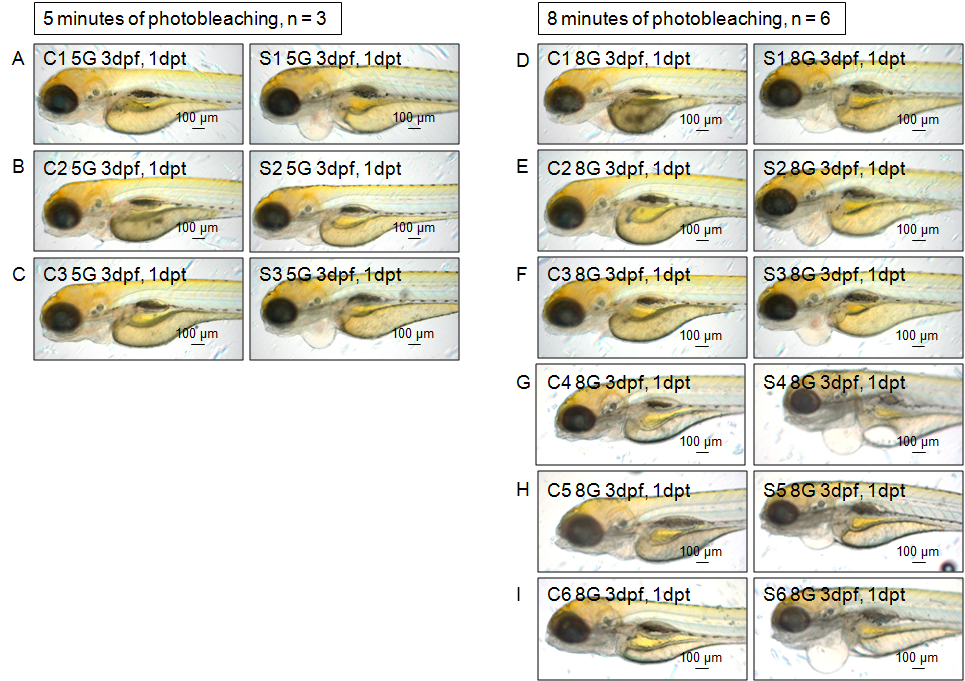

Supplement: Additional file 3 — Compilation of images of all illuminated SqKR15 larvae with pericardial edema a day after illumination. [file 1471-213X-10-110-S3.TIFF]

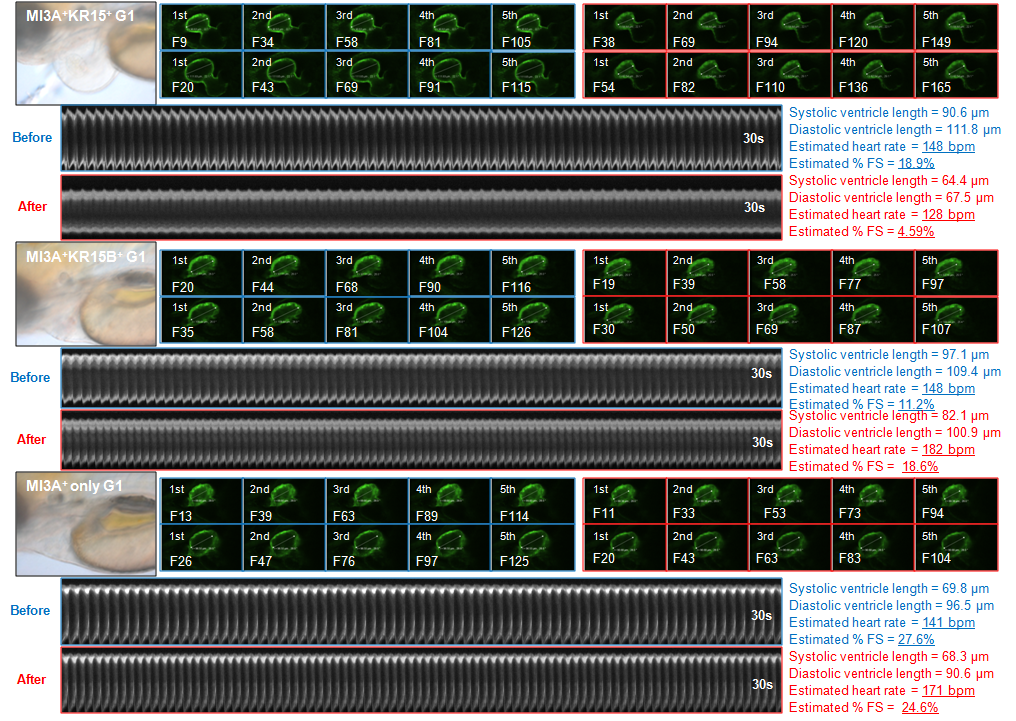

Supplement: Additional file 4 — First group of images used to generate the bar chart of percent change in heartbeat and contractility after illumination. Heart beat and contractility in SqKR15/ET33-mi3A (sample), SqKR15B/ET33-mi3A (skin control) and KR-negative Sq ET33-mi3A (negative control) 3dpf larvae before and 20 min after illumination. [file 1471-213X-10-110-S4.TIFF]

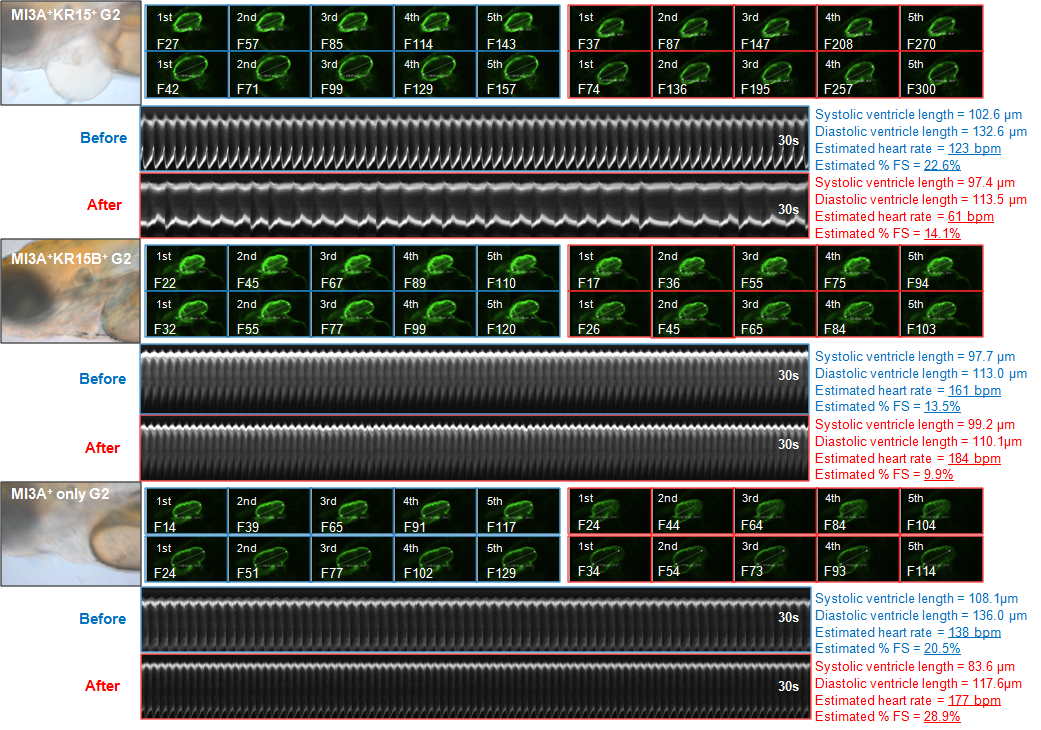

Supplement: Additional file 5 — Second group of images used to generate the bar chart of percent change in heartbeat and contractility after illumination. Heart beat and contractility in SqKR15/ET33-mi3A (sample), SqKR15B/ET33-mi3A (skin control) and KR-negative Sq ET33-mi3A (negative control) 3dpf larvae before and 20 min after illumination. [file 1471-213X-10-110-S5.TIFF]

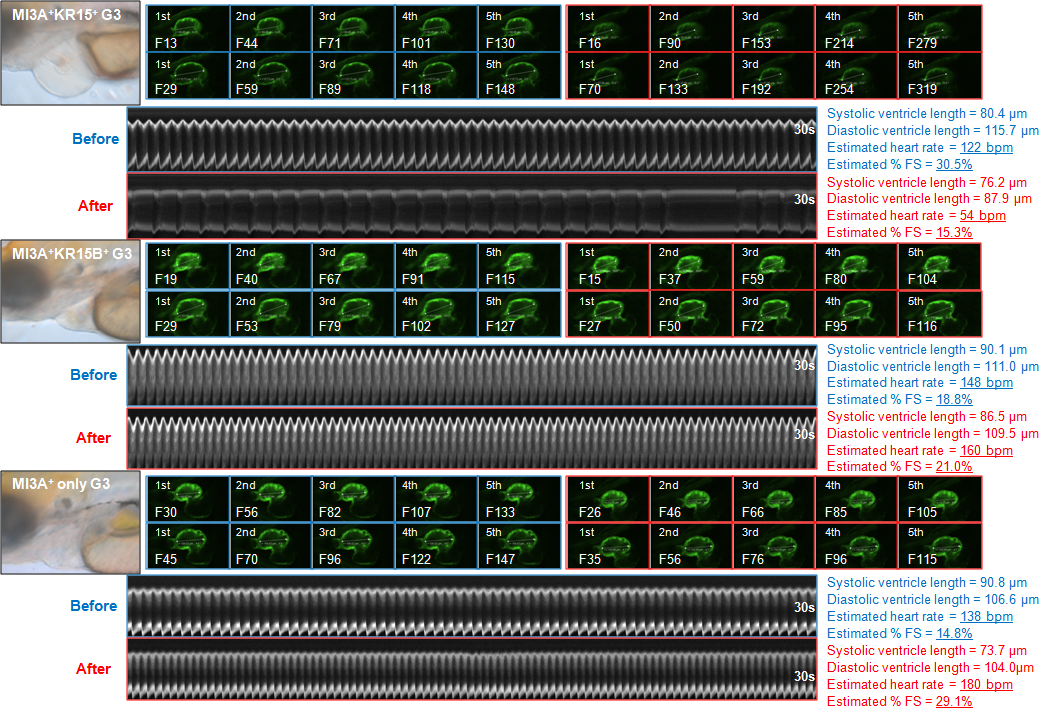

Supplement: Additional file 6 — Third group of images used to generate the bar chart of percent change in heartbeat and contractility after illumination. Heart beat and contractility in SqKR15/ET33-mi3A (sample), SqKR15B/ET33-mi3A (skin control) and KR-negative Sq ET33-mi3A (negative control) 3dpf larvae before and 20 min after illumination. [file 1471-213X-10-110-S6.TIFF]

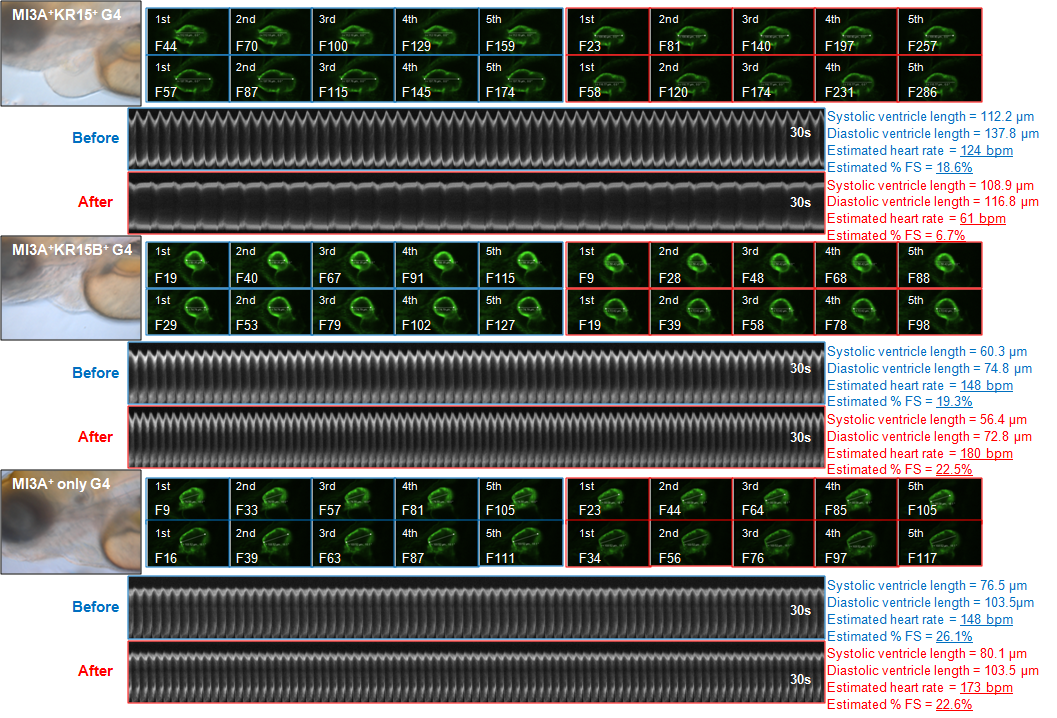

Supplement: Additional file 7 — Fourth group of images used to generate the bar chart of percent change in heartbeat and contractility after illumination. Heart beat and contractility in SqKR15/ET33-mi3A (sample), SqKR15B/ET33-mi3A (skin control) and KR-negative Sq ET33-mi3A (negative control) 3dpf larvae before and 20 min after illumination. [file 1471-213X-10-110-S7.TIFF]
